# Jasmonic Acid Enhances Rice Cadmium Tolerance by Suppressing Cadmium Uptake and Translocation

**DOI:** 10.3390/plants14071068

**Published:** 2025-03-31

**Authors:** Hao Zhang, Zhengkai Liu, Xinyu Li, Xiaodong Liu, Linzhi Fang, Rensen Zeng, Qiongli Wang, Yuanyuan Song, Daoqian Chen

**Affiliations:** 1 Key Laboratory of Ministry of Education for Genetics, Breeding and Multiple Utilization of Crops, College of Agriculture, Fujian Agriculture and Forestry University, Fuzhou 350002, China; 15864667335@163.com (H.Z.); liuzhengkai2022@163.com (Z.L.); lxyfafu@163.com (X.L.); 17352015063@163.com (X.L.); amazingseed@126.com (L.F.); rszeng@fafu.edu.cn (R.Z.); 2 Key Laboratory of Ministry of Agriculture and Rural Affairs of Biological Breeding for Fujian and Taiwan Crops, College of Agriculture, Fujian Agriculture and Forestry University, Fuzhou 350002, China; 3 College of Future Technology, Fujian Agriculture and Forestry University, Fuzhou 350002, China; 4 Shandong Branch of Sinochem Agriculture Holdings, Zibo 256304, China

**Keywords:** rice (*Oryza sativa* L.), Cd tolerance, jasmonic acid, Cd transporter

## Abstract

Worldwide, cadmium (Cd) contamination severely threatens rice production and public health. Jasmonic acid (JA) is recognized to be involved in rice Cd stress responses, but the underlying mechanism remains unclear. In this study, we show that JA positively regulates Cd tolerance in rice by repressing Cd uptake and root-to-shoot translocation. Cd exposure rapidly elevated the endogenous JA in rice roots, which was associated with increased expression of JA synthesis and JA-responsive genes. Moreover, silencing the expression of either *allene oxide synthase* (*OsAOS*; active in JA biosynthesis) or *CORONATINE INSENSITIVE1* (*OsCOI1*; active in JA perception) resulted in aggravated Cd toxicity and increased Cd accumulation in both the roots and shoots, as well as increased translocation from the root to the shoots. A short-term uptake experiment revealed that silencing of *OsAOS* and *OsCOI1* enhanced root Cd uptake ability. Furthermore, the elevated transcript levels of genes for Cd uptake (*OsNramp5*, *OsNramp1,* and *OsIRT1*) and root-to-shoot translocation (*OsHMA2*) were observed in *OsAOS* and *OsCOI1* RNAi plants in comparison with wild-type plants. Taken together, our findings suggest that JA enhances rice cadmium tolerance by suppressing Cd uptake and translocation.

## 1. Introduction

Heavy metal contamination has become a serious ecological concern worldwide, with the increasing release of heavy metals from anthropogenic activities [1,2]. Cadmium (Cd) is considered one of the most hazardous elements for both animals and plants for its high mobility and biotoxicity [3,4]. As a non-essential heavy metal, excessive amounts of soil Cd seriously inhibit plant growth and development by disturbing a series of physiological and biochemical processes, such as water and nutrient uptake, photosynthesis, transcription, and signaling pathways [5]. The accumulation of Cd in plants not only impairs plant growth, crop yield, and quality but also endangers human health via the food chain. Rice (*Oryza sativa* L.) is the staple food for half of the population of the world. However, Cd is almost ubiquitously present as an environmental pollutant in the rice-growing regions of the world [6,7]. Worse still is that rice appears to take up and accumulate Cd more than other major cereal crops. High Cd accumulation in rice leads to an excessive intake of toxic heavy metals by humans, posing a big threat to public health [2,8,9].

Cd in the soil could be readily absorbed by plant roots and then translocated to other tissues through a series of transporters [10,11]. Over the last decades, several key transporters involved in Cd uptake by the roots and root-to-shoot translocation have been identified in rice [11,12,13]. OsNramp5, a member of the natural resistance-associated macrophage protein family, is functionally characterized as the major transporter for Cd uptake from the soil solution to rice root cells. Knockout of this transporter results in a dramatic reduction in Cd and Mn uptake in rice [14]. In addition, OsNramp1, iron-regulated transporter OsIRT1, and the major facilitator superfamily protein OsCd1 also contribute to the uptake in rice roots [15,16,17]. Moreover, OsHMA2, a P-type heavy metal ATPase, is identified to be responsible for the root-to-shoot translocation of Cd. The knockout of OsHMA2 resulted in large decreases in the translocation of Cd from root to shoot [18,19].

Several signaling pathways, such as abscisic acid (ABA), jasmonic acid (JA), gibberellin, and auxin, have been suggested to be involved in the regulation of Cd stress responses [20,21,22,23]. However, their roles in affecting plant Cd uptake and translocation remain largely unclear. JA is an important phytohormone that is critical for growth, development, reproduction, and stress responses in plants [24,25]. The exposure of plants to Cd induces the expression of JA synthesis and JA-responsive genes, and/or increases endogenous JA levels in *Arabidopsis* [23,26]. Several studies have demonstrated that the exogenous application of JA could effectively alleviate Cd toxicity in *Arabidopsis* [23], *Capsicum frutescens* [27], *Solanum nigrum* [28], wheat [29], rice [30,31,32], etc. Recently, Wei et al. [31] reported that exogenous MeJA alleviated Cd toxicity, depressed Cd uptake, and modulated Cd distribution in rice seedlings. The decrease in Cd uptake and translocation by exogenous MeJA is correlated with the down-regulation of metal transporter genes in the roots [30,31]. These findings suggest a critical role for JA in controlling Cd uptake and translocation through the transcriptional regulation of the Cd transporter genes in rice.

In this study, we investigated the effect of Cd on JA synthesis in rice roots, and the transgenic RNAi lines of *allene oxide synthase* (*OsAOS*), which are involved in JA biosynthesis, and *CORONATINE INSENSITIVE1* (*OsCOI1*), which is involved in JA perception, were used to elucidate the effects of in vivo JA on Cd toxicity, Cd uptake, and translocation in rice [33].

## 2. Results

### 2.1. JA Synthesis Is Enhanced by Cd Exposure

**Figure 1:**
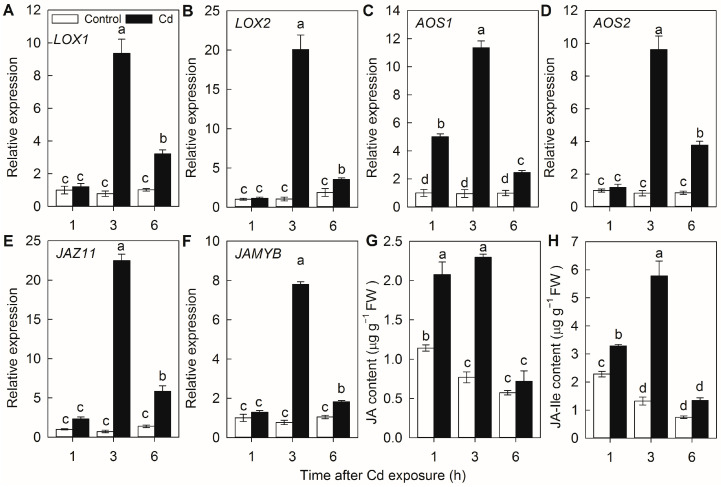
Effect of Cd exposure on JA levels in rice roots. Transcript levels of genes encoding lipoxygenases (*LOX1*, (**A**); *LOX2*, (**B**)), allene oxide synthases (*AOS1*, (**C**); *AOS2*, (**D**)), jasmonate ZIM-domain protein (*JAZ11*, (**E**)), JA-inducible Myb transcription factor (*JAMYB*, (**F**)). Contents of JA (**G**) and JA-Ile content (**H**) in rice plants treated with or without 50 μM CdCl_2_. Data are means ± SE (n = 3). Different letters above bars indicate statistically significant differences between treatments (Tukey’s multiple range test, *p* ≤ 0.05).

To determine whether Cd stress alters the endogenous JA levels, the transcript levels of key genes involved in JA biosynthesis, JA-responsive marker genes, and the accumulation of JA in rice roots exposed to Cd stress were monitored. The expression levels of genes encoding lipoxygenase (*LOX1* and 2) and allene oxide synthase (*AOS1* and 2), as well as jasmonate ZIM-domain protein (*JAZ11*) and JA-inducible Myb transcription factor (*JAMYB*), were rapidly induced by Cd exposure (Figure 1A–F). Such effect was particularly evident at 3 h after Cd treatment, at the time the transcript levels of the above genes were increased by 9.3, 20.1, 11.3, 9.6, 22.5, and 7.8 fold, respectively, relative to control plants without Cd treatment. Similarly, both JA and JA-Ile levels were significantly increased after Cd treatment (Figure 1G,H). These results indicate that Cd stress could rapidly enhance the JA accumulation.

### 2.2. Silencing of OsAOS and OsCOI1 Aggravates Cd Toxicity in Rice

**Figure 2:**
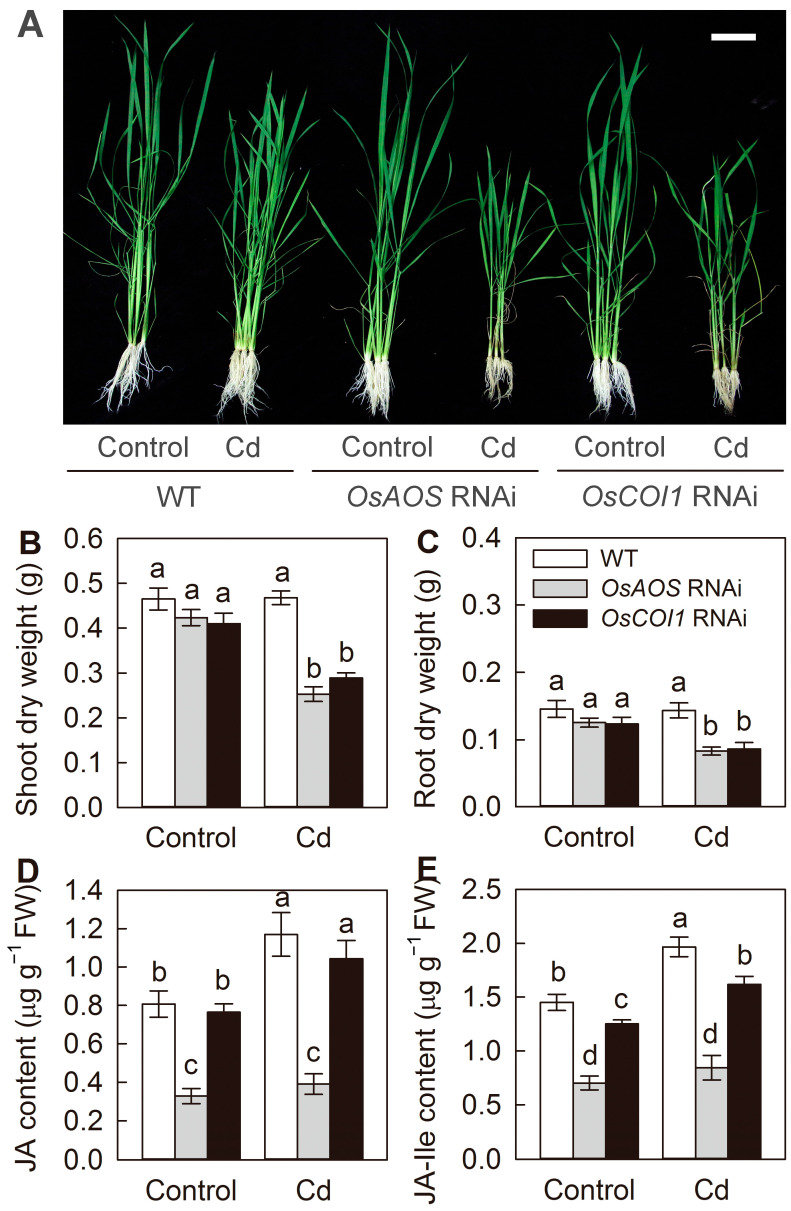
Effect of Cd exposure on growth of WT rice plants and the *OsAOS* and *OsCOI1* RNAi lines. Phenotype (**A**), dry weight of shoots (**B**) and roots (**C**), JA (**D**) and JA-Ile content (**E**) of rice plants treated with (Cd) or without (Control) 50 μM CdCl_2_ for 14 days. Bar, 10 cm. Data are means ± SE (n = 12). Different letters above bars indicate statistically significant differences between treatments (Tukey’s multiple range test, *p* ≤ 0.05).

**Figure 3:**
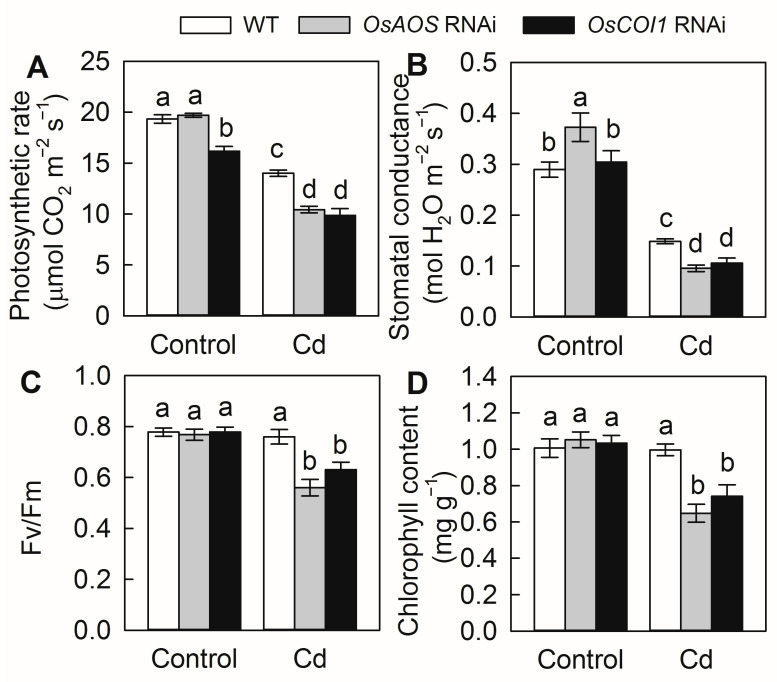
Effect of Cd exposure on photosynthesis of *OsAOS* and *OsCOI1* RNAi lines and corresponding WT rice plants. Photosynthetic rate (**A**), stomatal conductance (**B**), maximum efficiency of PSII photochemistry (Fv/Fm), (**C**) and chlorophyll content (**D**) of rice plants treated with (Cd) or without (Control) 50 μM CdCl_2_ for 14 days. Data are means ± SE (n = 6). Different letters above bars indicate statistically significant differences between treatments (Tukey’s multiple range test, *p* ≤ 0.05).

To further investigate the role of the JA pathway in the Cd stress response in vivo, the RNAi plants of *OsAOS* and *OsCOI1*, two critical genes involved in JA biosynthesis and perception, respectively, were exposed to Cd stress. Both *OsAOS* and *OsCOI1* RNAi plants showed significantly increased sensitivity to Cd stress. After 2 weeks of Cd exposure, the *OsAOS* and *OsCOI1* RNAi plants exhibited stronger growth inhibition and necrosis in the basal part of the stems (Figure 2A–C). In addition, both JA and JA-Ile levels decreased in *OsAOS* RNAi plants, while only JA-Ile levels slightly decreased in the *OsCOI1* RNAi plants under both control and Cd stress conditions (Figure 2D,E). Moreover, Cd exposure decreased the leaf photosynthetic rate and stomatal conductance of all three genotypes. However, the magnitudes of Cd-induced inhibition were significantly higher in RNAi plants (Figure 3A,B). The maximum efficiency of the PSII photochemistry (Fv/Fm) and chlorophyll content were significantly reduced by 2 weeks of Cd exposure in the *OsAOS* and *OsCOI1* RNAi plants, while Cd stress had no significant effect on them in the WT plants (Figure 3C,D). These results indicated that the decreased endogenous JA biosynthesis and perception aggravate Cd toxicity, and the JA signaling pathway is required for Cd tolerance in rice.

### 2.3. Silencing of OsAOS and OsCOI1 Increases Cd Accumulation

**Figure 4:**
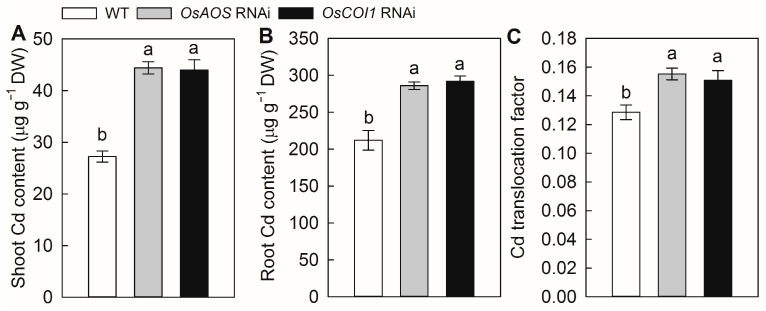
The effect of Cd stress on Cd accumulation and translocation of WT rice plants and the *OsAOS* and *OsCOI1* RNAi lines. Cd contents in the shoots (**A**) and roots (**B**), and root-to-shoot Cd translocation factor (**C**) of rice plants treated with 50 μM CdCl_2_ (Cd) for 7 days. Data are means ± SE (n = 4). Different letters above bars indicate statistically significant differences between treatments (Tukey’s multiple range test, *p* ≤ 0.05).

To test the potential effects of JA on Cd uptake and translocation, we determined the Cd accumulation in the rice seedlings of the three genotypes after 7 days of Cd exposure. The *OsAOS* and *OsCOI1* RNAi plants accumulated more Cd in both the shoots and roots compared with the WT plants (Figure 4A,B). After 7 days of Cd exposure, the shoot Cd contents of the *OsAOS* and *OsCOI1* RNAi plants were 63.0% and 61.7% higher than that in WT plants. And the root Cd contents of *OsAOS* and *OsCOI1* RNAi plants were 34.9% and 37.7% higher than that in WT plants. Meanwhile, the silencing of JA biosynthesis and perception also increased the root-to-shoot translocations, which were 20.8% and 17.4% higher than that of the WT plants. These results indicated that the JA signaling pathway is required for reducing root Cd uptake and Cd translocation from the root to shoot in rice.

### 2.4. Silencing of OsAOS and OsCOI1 Enhances Root Cd Uptake

**Figure 5:**
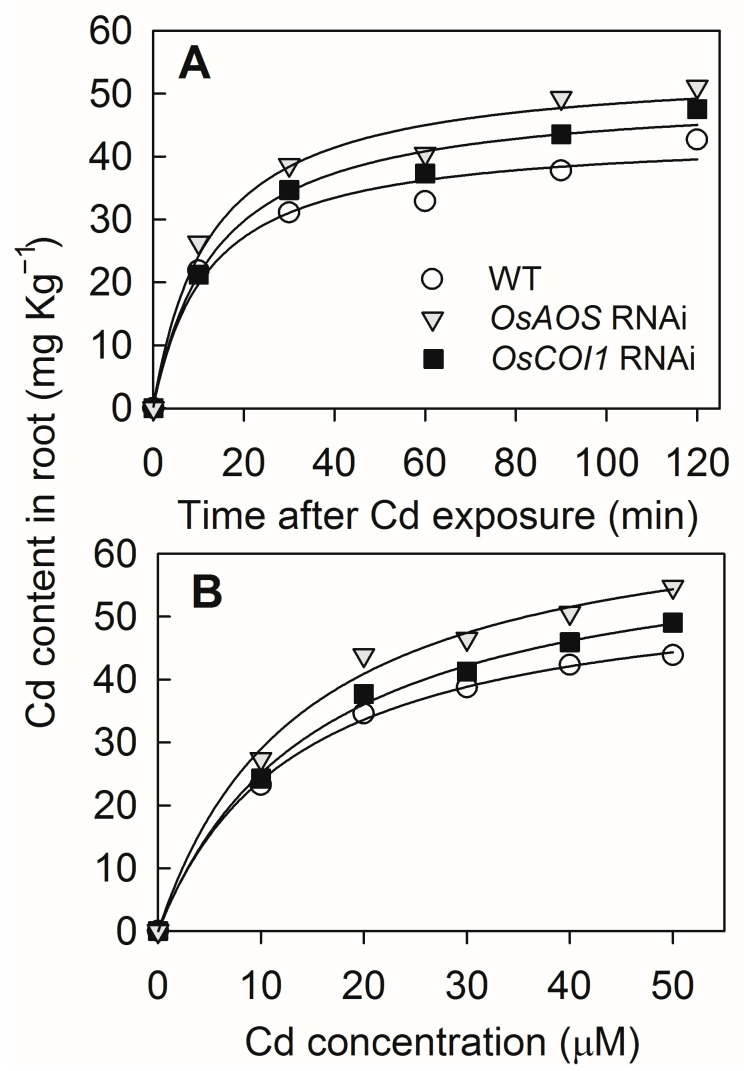
Short-term Cd uptake by roots of *OsAOS* and *OsCOI1* RNAi lines and corresponding WT rice plants. (**A**) Root Cd contents of rice plants treated with 50 μM CdCl_2_ for different times. (**B**) Root Cd contents of rice plants treated with different Cd concentrations at 25 °C for 30 min. Data are means ± SE (n = 4).

To confirm the role of JA on Cd uptake, the short-term root uptake experiments using the intact roots of the three genotypes were performed. In all three tested genotypes, the Cd uptake increased with time, from 0 to 120 min. However, the Cd uptake in the *OsAOS* and *OsCOI1* RNAi roots was faster than that in WT roots (Figure 5A). Similarly, the Cd uptake increased with the increasing Cd concentration in the nutrient solution in all three tested genotypes. But the Cd uptake in the *OsAOS* and *OsCOI1* RNAi roots was higher than that in the WT roots for all the exogenous Cd concentrations, except for 10 μM (Figure 5B). These results provided evidence that the JA signaling pathway is required for reducing root Cd uptake in response to Cd stress in rice.

### 2.5. Silencing of OsAOS and OsCOI1 Promotes Expression of Genes for Cd Uptake and Translocation

**Figure 6:**
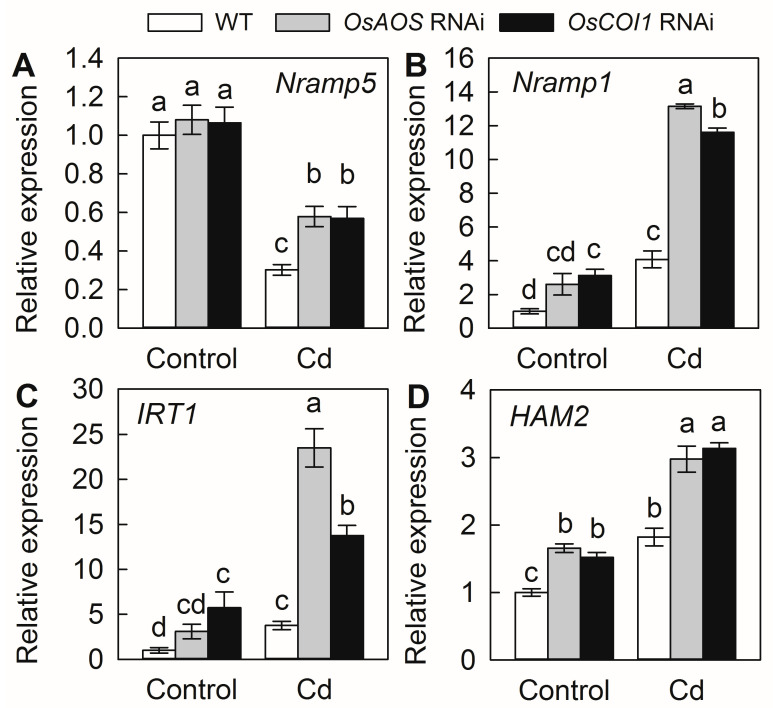
Effect of Cd exposure on expression of genes involved in Cd uptake and translocation in *OsAOS* and *OsCOI1* RNAi lines and corresponding WT rice roots. Transcript levels of genes encoding natural resistance-associated macrophage proteins (*Nramp5*, (**A**); *Nramp1*, (**B**)), iron-regulated transporter 1 (*IRT1*, (**C**)) and P-type heavy metal ATPase (*HMA2*, (**D**)) in rice roots treated with or without 50 μM CdCl_2_ for 7 days. Data are means ± SE (n = 3). Different letters above bars indicate statistically significant differences between treatments (Tukey’s multiple range test, *p* ≤ 0.05).

To explore how the JA pathway affects Cd uptake and translocation in rice plants, we analyzed the expression levels of the genes involved in Cd uptake and translocation by RT-qPCR. The transcript levels of *Nramp5* were repressed, while the expressions of *Nramp1*, *IRT1,* and *HMA2* were elevated by Cd exposure in all three tested genotypes (Figure 6). The mRNA levels of *Nramp1*, *IRT1,* and *HMA2* were all significantly higher in the *OsAOS* and *OsCOI1* RNAi roots than those in the WT in the absence of Cd. Silencing of the genes involved in JA biosynthesis and perception promoted the expression of all of the tested Cd transporter genes. These results indicate that endogenous JA negatively regulates the expressions of genes involved in Cd uptake and translocation from the roots to the shoots and, hence, decreases the Cd accumulation in rice plants.

## 3. Discussion

Cd contamination has emerged as one of the most important factors limiting rice production and threatening public health [2,17]. Over the last decades, intensive efforts have been made to identify the key transporters for Cd uptake and translocation in rice [11,17]. However, the precise regulatory signaling pathways affecting the Cd stress response, Cd uptake, and translocation in rice remain poorly understood. In the present study, our results demonstrated that Cd stress promotes rice endogenous JA synthesis, at least partially by the rapid induction of the expression of JA synthesis genes (Figure 1). Moreover, the silencing of the genes involved in either JA biosynthesis or perception resulted in higher Cd accumulation and increased sensitivity to Cd stress (Figure 2, Figure 3 and Figure 4). Therefore, our findings indicated that JA plays an important role in the Cd stress response, and the jasmonate signaling pathway is required for Cd tolerance in rice.

The crucial roles of the JA signaling pathway in mediating growth, development, and stress responses in plants have been investigated extensively [25]. It was previously demonstrated that exogenous application of MeJA could effectively alleviate Cd toxicity in Cd-stressed rice plants and reduce Cd-induced oxidative stress by enhancing the glutathione content and the activity of antioxidant enzymes [32]. Kanu et al. [34] reported that exogenous MeJA could alleviate Cd-induced oxidative stress, increase mineral nutrient uptake, and reduce Cd accumulation in rice plants. Yang et al. and Wei et al. [30,31] indicated that exogenous MeJA depressed Cd uptake and modulated Cd distribution by suppressing the expression of the Cd transporter genes in rice. Here, our results showed that the compromise of JA signaling by silencing of genes involved in JA biosynthesis and perception aggravates Cd toxicity in rice and increased Cd accumulation in both the roots and the shoots (Figure 2, Figure 3 and Figure 4). Furthermore, the short-term root uptake experiments proved that defectiveness in JA biosynthesis and perception enhances root Cd uptake activity (Figure 5). These results indicated that the JA signaling pathway regulates the Cd stress response by reducing root Cd uptake in rice. Similarly, the enhanced sensitivity to Cd stress has also been observed in the tomato JA deficiency mutant *spr2* [35], as well as the *Arabidopsis* JA synthesis mutant *ataos* and the JA receptor mutant *atcoi1* [23], suggesting that the regulatory role of JA signaling pathway in Cd stress response appears to be conserved across different plants species.

Although no specific transporters for Cd uptake and translocation have been found in rice so far, Cd could be easily absorbed by rice roots and translocated to other tissues by a number of other divalent metal transporters, such as Nramp5, Nramp1, HMA2, IRT1, etc. [10,11,17]. In the present study, the transcript levels of *Nramp5*, *Nramp1*, *IRT*1, and *HMA2* were all significantly promoted by the compromise of JA biosynthesis and perception, suggesting that JA negatively regulates the expression of genes involved in Cd uptake and translocation. Despite Nramp5 and HMA2 being identified as the major transporter responsible for uptake and root-to-shoot translocation of Cd, respectively, several other transporter and/or regulator genes have also been characterized to be involved in rice Cd uptake and accumulation, such as *Cd1*, *CCX2*, *ZIP5*, *ZIP9*, *HMA3*, *CAL1*, *ABCG36 LCT1*, etc. [9,10,11]. Thus, it is possible that the JA pathway might also regulate the expression of these genes to influence rice Cd uptake and translocation. Generally, the JA signaling could be rapidly activated by various stresses to regulate the expression of downstream JA-responsive genes [25]. Recently, it has been reported that JA negatively regulates iron acquisition under Fe deficiency conditions by activating the core JA signaling component MYC2, which promotes FIT (FER-like iron deficiency-induced transcription factor) degradation by regulating the expression of a set of bHLH transcription factors, resulting in a reduced expression of AtIRT1 in *Arabidopsis* [36]. Therefore, it is possible that Cd-induced JA might directly or indirectly repress the expression of genes mediating Cd uptake and root-to-shoot translocation to positively regulate the Cd response in rice. Further efforts on the functional characterization of the key components in the JA signal module mediating Cd uptake and translocation are required for future Cd resistance and/or low-Cd breeding in rice. In addition, compared with other plants, rice appears to display high tolerance to Cd toxicity, and researchers have traditionally used excessively high Cd concentrations to investigate the responses of rice to Cd stress [23,30,31,32]. In the present study, the WT seedlings did not exhibit an abnormal growth phenotype, except for a slight decrease in plant height and necrosis in the basal part of stems, associated with unaffected Fv/Fm and chlorophyll content after a 2-week-long 50 µM Cd treatment (substantially high dose) (Figure 2 and Figure 3). However, Cd concentrations in soil solutions typically remain below 1 μM, even in heavily contaminated areas near smelters [2]. Therefore, further efforts on the potential role of JA in regulating Cd uptake and accumulation in rice grains under realistic Cd concentrations will provide more direct guidance for low-Cd breeding in rice.

## 4. Materials and Methods

### 4.1. Plant Materials and Growth Conditions

The rice (*Oryza sativa* L.) *OsAOS* and *OsCOI1* RNAi lines, as well as their corresponding wild type (cv. Ishikari-Shiroke, WT), were used in this study [33]. Rice seeds were surface sterilized and transferred to a seeding tray for germination. The 7-day-old seedlings were transplanted into a plastic box containing 5 L full-strength modified Kimura B nutrient solution (pH: 5.6, renewed every 3 d), as described by Chen et al. [8]. The rice plants were cultivated in a growth chamber under a 12 h/12 h day/night cycle, with the temperature regime at 27 °C/23 °C, and a light intensity of 300 μmol m^−2^ s^−1^. After 4 weeks, the uniform healthy rice seedlings were treated with (Cd) or without (Control) 50 μM CdCl_2_. To examine the effect of Cd exposure on rice growth, 12 seedlings were harvested after 14 days of Cd treatment and dried at 75 °C for 3 d to measure the shoot and root dry weight.

### 4.2. Leaf Gas Exchange Analysis

The photosynthetic rates and stomatal conductance of the youngest fully expanded leaves were measured between 9:00 and 11:00 a.m. using a portable photosynthesis system (Li-6400; LI-COR Inc., Lincoln, NE, USA) with a 6 cm^2^ chamber, and the photo flux density was set to 500 µmol m^−2^ s^−1^. Each treatment included six replicates.

### 4.3. Chlorophyll Fluorescence Analysis

The photosystem II photochemistry efficiency (Fv/Fm) of the youngest fully expanded leaves was analyzed after dark adaptation for 30 min by a pulse amplitude-modulated chlorophyll fluorescence system (Imaging PAM, Walz, Effeltrich, Germany), according to the method of Chen et al. [37]. Chlorophyll content was determined by a spectrophotometer (UV-2550, Shimadzu, Kyoto, Japan) after extraction with 80% ice-cold acetone. Each treatment included six replicates.

### 4.4. JA and JA-Ile Analysis

Frozen leaves were used for the quantification of JA and JA-Ile by UPLC-MS/MS, as described by Song et al. [38]. In brief, the fine powder of leaf tissues (approximately 200 mg) was extracted with 1 mL of ice-cold ethyl acetate spiked with the internal standards (20 ng of D6-JA and 5 ng of ^13^C6-JA-Ile). After homogenization by vortexing for 10 min and centrifugation at 13,000 *g* for 10 min, the supernatants were transferred to 2 mL tubes for vacuum evaporation. Then, the pellets were resuspended in 0.2 mL of 50% (*v*/*v*) methanol and centrifuged at 13,000 *g* for 15 min. The supernatant was transferred to glass vials and then subjected to HPLC-MS/MS analysis (LCMS-8040, Shimadzu, Kyoto, Japan). The JA and JA-Ile contents were quantified by comparing their peak area with the peak area of their respective internal standard. Each treatment included three replicates.

### 4.5. Cd Content Measurement

Dried rice shoot and root samples were milled to a fine powder for Cd content analysis. The powder was weighed (approximately 500 mg) and digested in nitric acid (GR 65.0–68.0%) in a glass tube at 340 °C on a hot stove, and 20 μL of H_2_O_2_ (GR ≥ 30.0%) was added 2–3 times during the digestion. The Cd concentration in the supernatant was then determined using atomic absorption spectrometry (Zeenit700p, Analytik Jena, Jena/Überlingen, Germany). Data were expressed on the basis of dry weight.

### 4.6. Short-Term Cd Uptake Determination

To examine Cd transport activity in plants, we performed short-term uptake experiments using intact plants of both wild-type rice and the RNAi lines. First, the rice seedlings (4 weeks old) were subjected to an uptake solution containing 50 μM CdCl_2_ for different times (0, 10, 30, 60, 90, and 120 min) at 25 °C. Meanwhile, the rice seedlings (4 weeks old) were subjected to an uptake solution containing various concentrations of Cd (0, 10, 20, 30, 40, and 50 μM) at 25 °C for 30 min. Then, the roots were separated, dried at 75 °C for 3 d, and used for Cd content determination, as described before.

### 4.7. Gene Expression Analysis

For gene expression analysis, frozen root samples (approximately 100 mg) were used for RNA extraction. The gene expression analysis was conducted using a quantitative RT-PCR, as previously described by Chen et al. [8], with *OsActin* as the internal reference gene. The primer specificity was validated through primer-BLAST and verified by a melt curve analysis. The 2^−∆∆Ct^ method was used for the relative expression calculation. All experiments were performed in triplicate using three biological replicates per treatment. The gene-specific primers are listed in Table S1.

### 4.8. Statistical Analysis

Statistical analysis was performed using the SPSS statistics software (Version 19.0 for Windows, SPSS, Chicago, IL, USA). The data were subjected to an analysis of variance (ANOVA) with Tukey’s test for differences among treatments, and a *p* value ≤ 0.05 was considered significant between the treatments. All values are presented as the mean ± SE. Graphs were generated using GraphPad Prism 8.0 (GraphPad Software Inc., La Jolla, CA, USA).

## 5. Conclusions

Our present study reveals that the exposure of rice plants to Cd stress rapidly activates the JA signaling pathway, which positively regulates the Cd response by repressing the transcriptional levels of the genes mediating Cd uptake and root-to-shoot translocation. Our findings provide direct evidence for the in vivo effects of JA on Cd toxicity, Cd uptake, and translocation and highlight the potential of JA signaling in enhancing Cd resistance in rice.

## Data Availability

Data are contained within the article and Supplementary Materials.

## Supplementary Materials

The following supporting information can be downloaded at https://www.mdpi.com/article/10.3390/plants14071068/s1: Table S1: Primers used in the real-time quantitative PCR experiment.

## Abbreviations

The following abbreviations are used in this manuscript:

Cd	Cadmium
JA	Jasmonic acid
RNAi	RNA interference
WT	Wild type
AOS	Allene oxide synthase
COI	CORONATINE INSENSITIVE
LOX	Lipoxygenase
IRT	iron-regulated transporter
Nramp	Natural resistance-associated macrophage protein
HMA	P-type heavy metal ATPase
RT-qPCR	Reverse transcription–quantitative PCR
