# Supplementary material for: Jasmonic Acid Enhances Rice Cadmium Tolerance by Suppressing Cadmium Uptake and Translocation
Source: Plants (Basel). 2025 Mar 31;14(7):1068. doi: 10.3390/plants14071068 (PMC11991435; doi:10.3390/plants14071068)
Supplement: Supplementary file 1 [file plants-14-01068-s001.zip › plants-3539996-supplementary.pdf]

Table S1. Primers used in the real-time quantitative PCR experiment.

Gene	Loci	Primers (5'-3')
OsActin	*Os03g50885*	5'-GACTCTGGTGATGGTGTCAGC-3'
		5'-GACTCTGGTGATGGTGTCAGC-3'
OsLOX1	*Os02g0194700*	5'-GTACGCTGGGTTCACAGCTC-3'
		5'-TTCAGATGGATGTGCTGTTGG-3'
OsLOX2	*Os03g0179900*	5'-GCATCCCCAACAGCACATC-3'
		5'-AATAAAGATTTGGGAGTGACATATTGG-3'
OsAOS1	*Os03g0767000*	5'-CGGGACATGGTGGTGGAGA-3'
		5'-GGAGTCGTATCGGAGGAAGAGC-3'
OsAOS2	*Os03g0225900*	5'-GAGGACCAGAGTGCAAAAGC-3'
		5'-CATGGCATCCTTGAACCTCT-3'
OsJAZ11	*Os03g0180900*	5'-AGTACATGAAGGAGCACAGTG-3'
		5'-CTTCCTTTCTTGCGTGCTTTC-3'
OsJAMyb	*Os11g0684000*	5'-AGTACATGAAGGAGCACAGTG-3'
		5'-CTTCCTTTCTTGCGTGCTTTC-3'
OsNramp1	*Os07g0258400*	5'-CATGTCCGTCATGGCCAAGT-3'
		5'-TGTCTGCAGCTGATGATCGAG-3'
OsNramp5	*Os07g0257200*	5'-CAGCAGCAGTAAGAGCAAGATG-3'
		5'-GTGCTCAGGAAGTACATGTTGA-3'
OsIRT1	*Os03g0667500*	*5'-CGTCTTCTTCTTCTCCACCACGAC-3'*
		5'-GCAGCTGATGATCGAGTCTGACC-3'
OsHMA2	*Os06g0700700*	*5'-CATAGTGAAGCTGCCTGAGATC-3'*
		5'-GATCAAACGCATAGCAGCATCG-3'
